# Structural flexibility and heterogeneity of recombinant human glial fibrillary acidic protein

**DOI:** 10.1002/alz.083589

**Published:** 2025-01-09

**Authors:** Dea Gogishvili

**Affiliations:** ^1^ Vrije Universiteit Amsterdam, Amsterdam, North Holland Netherlands

## Abstract

**Background:**

Glial fibrillary acidic protein (GFAP) is a promising biomarker for brain and spinal cord disorders. Recent studies have highlighted the differences in the reliability of GFAP measurements in different biological matrices. The reason for these discrepancies is poorly understood as our knowledge of the protein's 3‐dimensional conformation, proteoforms, and aggregation remains limited.

**Method:**

Here, we investigate the structural properties of GFAP under different conditions. For this, we characterised recombinant GFAP proteins from various suppliers and applied hydrogen‐deuterium exchange mass spectrometry (HDX‐MS) to provide a snapshot of the conformational dynamics of GFAP in artificial cerebrospinal fluid (aCSF) compared to the phosphate buffer.

**Result:**

Our findings indicate that recombinant GFAP exists in various conformational species. Furthermore, we show that GFAP dimers remained intact under denaturing conditions. HDX‐MS experiments show an overall decrease in H‐bonding and an increase in solvent accessibility of GFAP in aCSF compared to the phosphate buffer, with clear indications of mixed EX2 and EX1 kinetics. To understand possible structural interface regions and the evolutionary conservation profiles, we combined HDX‐MS results with the predicted GFAP‐dimer structure by AlphaFold‐Multimer. We found that deprotected regions with high structural flexibility in aCSF overlap with predicted conserved dimeric 1B and 2B domain interfaces.

**Conclusion:**

Structural property predictions combined with the HDX data show an overall deprotection of GFAP in aCSF and signatures of aggregation. We anticipate that the outcomes of this research will contribute to a deeper understanding of the structural flexibility of GFAP and ultimately shed light on its behaviour in different biological matrices.

